# Honokiol Protects the Kidney from Renal Ischemia and Reperfusion Injury by Upregulating the Glutathione Biosynthetic Enzymes

**DOI:** 10.3390/biomedicines8090352

**Published:** 2020-09-15

**Authors:** Eun Jung Park, Theodomir Dusabimana, Jihyun Je, Kyuho Jeong, Seung Pil Yun, Hye Jung Kim, Hwajin Kim, Sang Won Park

**Affiliations:** 1 Department of Pharmacology, Institute of Health Sciences, Gyeongsang National University College of Medicine, Jinju 52727, Korea; foreverpak1@nate.com (E.J.P.); odomy2020@gmail.com (T.D.); jeri1984@naver.com (J.J.); khjeong@gnu.ac.kr (K.J.); spyun@gnu.ac.kr (S.P.Y.); hyejungkim@gnu.ac.kr (H.J.K.); 2 Department of Convergence Medical Sciences, Institute of Health Sciences, Gyeongsang National University Graduate School, Jinju 52727, Korea

**Keywords:** acute kidney injury, antioxidant, glutamate-cysteine ligase, glutathione, honokiol, nuclear factor-erythroid 2 related factor 2, proximal tubule, renal ischemia reperfusion

## Abstract

Glutathione (GSH) is an endogenous antioxidant found in plants, animals, fungi, and some microorganisms that protects cells by neutralizing hydrogen peroxide. Honokiol, an active ingredient of *Magnolia officinalis*, is known for antioxidant, anti-inflammatory, and anti-bacterial properties. We investigated the protective mechanism of honokiol through regulating cellular GSH in renal proximal tubules against acute kidney injury (AKI). First, we measured cellular GSH levels and correlated them with the expression of GSH biosynthetic enzymes after honokiol treatment in human kidney-2 (HK-2) cells. Second, we used pharmacological inhibitors or siRNA-mediated gene silencing approach to determine the signaling pathway induced by honokiol. Third, the protective effect of honokiol via *de novo* GSH biosynthesis was investigated in renal ischemia-reperfusion (IR) mice. Honokiol significantly increased cellular GSH levels by upregulating the subunits of glutamate-cysteine ligase (Gcl)—Gclc and Gclm. These increases were mediated by activation of nuclear factor erythroid 2-related factor 2, via PI3K/Akt and protein kinase C signaling. Consistently, honokiol treatment reduced the plasma creatinine, tubular cell death, neutrophil infiltration and lipid peroxidation in IR mice and the effect was correlated with upregulation of Gclc and Gclm. Conclusively, honokiol may benefit to patients with AKI by increasing antioxidant GSH via transcriptional activation of the biosynthetic enzymes.

## 1. Introduction

Ischemia-reperfusion (IR) injury is the tissue damage caused by an ischemic period lacking oxygen and nutrients by blood blockage, followed by a sudden restoration of blood flow resulting in severe oxidative and inflammatory stress reactions. Thus, IR injury is more serious than ischemia alone, due to the burst of reactive oxygen species, leading to endothelial and organ dysfunction in various clinical conditions [1]. The pathophysiology of kidney IR is complicated, but reactive oxygen species (ROS), inflammatory mediators, and dysfunctional mitochondria have been shown to be involved [2].

The reperfusion of ischemic tissue creates an imbalance between the rate of ROS generation and the tissue’s ability to detoxify ROS. The major sources of excessive ROS production are xanthine oxidase (XO), NADPH oxidase (Nox), mitochondria, and uncoupled nitric oxide synthase (NOS) [3]. The kidney tissues exposed IR presented a substantial accumulation of XO substrates (hypoxanthine, xanthine, and succinate) [4] and the XO inhibitors (e.g., febuxostat, allopurinol) suppressed renal IR injury via reduced oxidative stress [5,6]. Nox2 and Nox4 were major enzymes to produce ROS and their expression and activity were altered in renal IR [7,8,9]. The mitochondrial electron transport chain (ETC) plays a dominant role in IR injury, due to uncoupling of ETC and subsequent electron leakage [10], and mitochondria-targeted antioxidants (e.g., MitoQ, SkQ1, etc.) are shown to protect the kidney from IR and may be promising for clinical use [11].

Under IR, oxidative stress is also caused by reduced or impaired endogenous antioxidant activities [12]. There are multiple antioxidant systems to protect the tissue from oxidative stress, which include superoxide dismutases (SODs), catalase and peroxidases, physiological cofactors (e.g., cysteine and glutathione (GSH)), heme oxygenase-1 (HO-1), and dietary antioxidants (e.g., ascorbate, tocopherol) [13]. GSH is a tripeptide, γ-L-glutamyl-cysteinyl-glycine, and a pivotal antioxidant found in plants, animals, fungi, and some microorganisms. GSH protects cellular components by neutralizing ROS, hydrogen peroxide, and prevents from lipid peroxidation and the ROS-induced protein and DNA damage [14]. Most of cellular GSH is present in the cytosol, and readily oxidized to glutathione disulfide (GSSG) by ROS, and thus the [GSH]:[GSSG] ratio is often used as an indicator of cellular redox state [15]. Two enzymes, glutamate-cysteine ligase (Gcl) and glutathione synthetase (Gss) act sequentially for GSH synthesis. Gcl, the rate-limiting enzyme of GSH synthesis, is a heterodimer consisting of catalytic and modulatory subunits, Gclc and Gclm, respectively; whereas, Gss is a homodimer [15]. The catalytic Gclc subunit is necessary and sufficient for the enzymatic activity, whereas the modulatory Gclm subunit increases the catalytic efficiency; thus, mice lacking Gclc are embryonic lethal [16]. Mice lacking Gclm have no overt phenotype; but are markedly reduced in GSH and susceptible to oxidative insults [17,18,19]. Gcl expression is inducible to oxidative stress and has shown to be regulated by nuclear factor-erythroid 2 related factor 2 (Nrf2), activator protein-1 (AP-1) and nuclear factor κB (NFκB) [20]. The overall rate of GSH synthesis is controlled by the substrate availability, amount of two subunits of Gcl, and extent of feedback inhibition by GSH and ATP [21]. The downregulation of GSH was shown to contribute to oxidative stress in rats exposed to renal IR [22,23], and thus, therapeutic strategies to increase GSH biosynthesis are of a great attention as effective antioxidants.

Nrf2 is a key transcription factor involved in cellular responses against oxidative stress, and regulates many cytoprotective genes in GSH production, ROS detoxification, NADPH regeneration, and heme and iron metabolism [24]. Under a basal condition, Nrf2 is suppressed by binding to Kelch-like ECH-associated protein 1 (keap1) and rapidly degraded by proteasomes in cytoplasm. Under stressed conditions, Nrf2 dissociated from keap1 is stabilized, and translocated into nucleus, and binds to a *cis*-acting antioxidant response element (ARE) that activate transcription of antioxidant genes [25,26]. Nrf2 KO mice were vulnerable to various chemical toxicities and diseases associated with oxidative pathology [27], whereas pharmacological activation of Nrf2 protects from diseases underlined by oxidative stress and inflammation [28].

Honokiol is an active ingredient of *Magnolia officinalis*, extensively used for traditional Asian medicine, and has known for its antioxidant, anti-inflammatory, anti-cancer, and anti-bacterial properties in various diseases. Honokiol has been shown to protect against renal IR and sepsis-induced acute kidney injury through inhibition of oxidative stress and inflammation [29,30], but the underlying molecular mechanisms of antioxidant properties have not been clearly shown. Here, we investigated whether honokiol has a modulatory effect on the biosynthesis of GSH during renal IR injury in vivo and the molecular mechanisms were elucidated using chemical inhibitors and siRNA-mediated gene silencing in proximal tubular human kidney-2 (HK-2) cells in vitro.

## 2. Experimental Section

### 2.1. Animals

Male C57BL/6 mice (7-weeks old) were purchased from Koatech (Pyeongtaek, South Korea) and maintained in the animal facility at Gyeongsang National University (GNU). All animal experiments were approved by the Institutional Board of Animal Research at GNU (GNU-180615-M0028) and performed in accordance with the National Institutes of Health guidelines for laboratory animal care. Mice were housed with an alternating 12-h light/dark cycle and provided with water and standard chow (Harlan Laboratories, Indianapolis, IN, USA) ad libitum. 

### 2.2. Animal Model of Renal IR

Mice were divided into four groups: (1) sham-operated mice treated with vehicle (Veh sham, *n* = 4), (2) mice treated with vehicle and subjected to bilateral renal IR (Veh IR, *n* = 8), (3) mice treated with honokiol (Sigma-Aldrich, St. Louis, MA, USA) and subjected to bilateral renal IR (HNK IR, *n* = 8), and (4) sham-operated mice treated with honokiol (HNK sham, *n* = 4). The mice were anesthetized with zoletil (0.5 mg/kg; Virbac Laboratories, Carros, France) and placed supine on a heating pad under a heat lamp to maintain body temperature at 37 °C. Kidneys were exposed, and the right and left renal pedicles were clamped with microaneurysm clips. After 25 min of ischemia, the clips were removed to allow reperfusion, and abdomen was closed by suture. Honokiol (1 mg/kg) was dissolved in a mixture of dimethyl sulfoxide (Sigma-Aldrich), Tween-20 (Sigma-Aldrich), and water at 1:1:8 ratio, and intraperitoneally injected twice, 1 h prior to ischemia and 4 h after reperfusion. Mice were sacrificed 24 h after reperfusion and the blood and kidney tissues were collected. 

The excised kidney was immediately fixed in 10% formalin (Sigma-Aldrich) for histology or snap-frozen in liquid nitrogen and stored at −80 °C for biochemical analysis. Plasma creatinine was measured by a direct colorimetric Jaffe method and detected by using a spectrophotometer (Shimadzu UV-1800 spectrophotometer, Tokyo, Japan), as previously described [31].

### 2.3. H&E Staining and Immunohistochemistry (IHC) 

The formalin-fixed kidney was processed for paraffin embedding. Five µm-thick paraffin sections were prepared and stained with hematoxylin and eosin (H&E) (Sigma-Aldrich). All images were captured by using a CKX41 light microscope (Olympus, Tokyo, Japan).

For immunohistochemistry (IHC) analysis, the sections were deparaffinized, rehydrated, and antigen-retrieved in sodium citrate buffer (10 mM, pH 6.0; iNtRON Biotechnology, Seongnam, Korea) for 20 min. The sections were blocked in 10% normal horse serum (Vector Laboratories, Burlingame, CA, USA) and incubated with a primary antibody for Ly-6B.2 (Bio-Rad, Hercules, CA, USA) or 4-hydroxynonenal (4-HNE; Abcam, Cambridge, UK) overnight at 4 °C. Sections were washed and incubated with a biotinylated secondary antibody (Vector Laboratories) for 1 h at room temperature. Sections were washed again and incubated in avidin-biotin-peroxidase complex solution (ABC solution; Vector Laboratories) and then developed using a 3,3′-diaminobenzidine (DAB) Peroxidase Substrate Kit (Vector Laboratories). The sections were counterstained with hematoxylin and analyzed using a CKX41 light microscope (Olympus).

### 2.4. Terminal Deoxynucleotidyl Transferase dUTP Nick End Labeling (TUNEL) Assay

Terminal deoxynucleotidyl transferase dUTP nick end labeling (TUNEL) assay was performed using an in situ cell death detection kit (Roche Molecular Biochemicals, Mannheim, Germany) according to the manufacturer’s protocol. Briefly, kidney sections were deparaffinized, and permeabilized with proteinase K (Abcam) at room temperature for 15 min. The sections were incubated with the labeling reaction mixture at 37 °C for 60 min. After washing with the PBS, the sections were mounted with ProLong Gold antifade reagent with DAPI (Invitrogen, Carlsbad, CA, USA). Images were captured using a CKX41 light microscope (Olympus) and quantified by using Image J (NIH, Bethesda, MD, USA). 

### 2.5. Cell Culture and Treatment

Human proximal tubular epithelial human kidney-2 (HK-2) cells were obtained from the ATCC (Manassas, VA, USA) and maintained in a 1:1 mixture of Dulbecco’s modified Eagle medium (Thermo Fisher Scientific, Waltham, MA, USA)/Kaighn’s modification of Ham’s F-12 medium (F-12K; Thermo Fisher Scientific), supplemented with 10% fetal bovine serum and 1% penicillin/streptomycin (Hyclone Laboratories, Logan, UT, USA). The cells were incubated at 37 °C in a 5% CO_2_ and 95% air humidified chamber (Forma 310 Direct Heat CO_2_ Incubator; Thermo Fisher Scientific). 

### 2.6. siRNA-Mediated Transfection

HK-2 cells were transfected with Nrf2, PKCα, PKCβ, or PKCδ specific siRNA along with control siRNA (Bioneer, Daejeon, Korea) using the Lipofectamine RNAiMAX (Invitrogen) at 50 or 100 nM of siRNA. After incubation for 24 h, honokiol, LY294002, wortmannin, or Gö6983 (Sigma-Aldrich) were treated as indicated in figure legends.

### 2.7. Cell Viability Assay 

Cell viability was determined by 3-(4,5-dimethylthiazol-2yl)-2,5-diphenyltetrazolium bromide (MTT; Sigma-Aldrich) assay. MTT solution (final 0.1 mg/mL) was added to each well, and cells were incubated for 4 h at 37 °C. The supernatants were aspirated, the formazan crystals in each well were dissolved in 200 μL of dimethyl sulfoxide. Absorbance was measured at 570 nm using an Infinite 200 PRO microplate reader (Tecan, Mannedor, Switzerland).

### 2.8. GSH Measurement

The GSH contents were measured using a GSH/GSSG ration detection kit II (Abcam) according to the manufacturer’s instruction. The cells were washed with PBS and collected in RIPA buffer (Thermo Fisher Scientific). After centrifugation, samples were deproteinized using trichloroacetic acid (Sigma-Aldrich)/sodium bicarbonate (NaHCO_3_; Sigma-Aldrich) to remove enzymes that interfere with the analysis. Samples were added to the assay mixture at room temperature for 10–60 min protected from light. Fluorescence at Ex/Em = 490/520 nm was measured using an Infinite 200 PRO microplate reader (Tecan).

### 2.9. Lipid Peroxidation (Malondialdehyde, MDA) Assay

MDA assay was performed using a Malondialdehyde (MDA) assay kit (Abcam) according to the manufacturer’s protocol. Briefly, the cells were washed with PBS and collected in RIPA buffer (Thermo Fisher Scientific). After centrifugation, TBA solution was added to samples, and the TBA-sample mixtures were incubated at 95 °C for 60 min and put on ice for 10 min for cooling. Absorbance was measured at 532 nm using an Infinite 200 PRO microplate reader (Tecan). 

### 2.10. Antioxidant Response Element (ARE)-Luciferase Reporter Assay

Cells were transfected with 1 μg of the luciferase reporter gene fusion construct (pTi-luciferase) with a wild type ARE sequence and 0.5 μg of the pCMV-β-galactosidase internal control vector with Lipofectamine 2000 (Invitrogen) according to the manufacturer’s instructions. After incubation for 24 h, the cells were treated with honokiol for additional 4 h. Then, the cells were lysed in 1× reporter lysis buffer (Promega, Madison, WI, USA). After mixing the cell lysates with a luciferase substrate (Promega), the luciferase activity was measured by a TD-20/20 luminometer (Turner Designs, San Jose, CA, USA) according to the manufacturer’s instructions.

### 2.11. Western Blot Analysis

Kidney tissues or HK-2 cells were homogenized in ice-cold RIPA buffer (Thermo Fisher Scientific) containing protease inhibitors (Thermo Fisher Scientific), sonicated, and incubated for 20 min on ice. After centrifugation, the supernatant was saved and protein concentration was determined using the Bradford assay (Bio-Rad). Nuclear and cytoplasmic fractionations were conducted by using NE-PERTM Nuclear and cytoplasmic extraction reagents (Thermo Fisher Scientific) according to the manufacturer’s instructions.

The protein samples were electrophoresed on polyacrylamide gels, and transferred to PVDF membranes (Roche, Basel, Switzerland). After blocking, the membranes were incubated with primary antibodies against Gclc, Gclm, Gss, and p84 (Abcam); Nrf2, p-Akt, p-p38, p-JNK, p-ERK, p-PKC (pan), p-PKCα/βII, and p-PKCδ (Cell Signaling Technology, Danvers, MA, USA); β-actin and β tubulin (Sigma-Aldrich) at 4 °C overnight. The membranes were incubated with horseradish peroxidase-conjugated secondary antibodies (Bio-Rad) at RT for 1 h. The membranes were incubated with Electrochemiluminescence (ECL) substrates (Bio-Rad) and protein bands were visualized using the ChemiDoc XRS+ System (Bio-Rad). 

### 2.12. Reverse Transcription and Quantitative PCR (qPCR) Analysis

Total RNA was extracted using Trizol (Invitrogen) and converted into cDNA with the RevertAid Reverse Transcription System (Thermo Fisher Scientific), according to the manufacturer’s instructions. Quantitative PCR was performed on the CFX Connect Real-Time PCR System using iQ SYBR Green Supermix (Bio-Rad). Relative mRNA levels were normalized to those of GAPDH for each gene. The primer sequences are listed in Table 1. 

### 2.13. Statistical Analysis

Statistical difference was determined using SigmaPlot software (Systat Software, Inc., San Jose, CA, USA). Comparisons among the groups were conducted by one-way analysis of variance (ANOVA), followed by Newman–Keuls tests. Data are expressed as the mean ± SEM. A *p* < 0.05 was accepted as statistically significant.

## 3. Results

### 3.1. Honokiol Treatment Increased GSH Levels in HK-2 Cells

**Figure 1:**
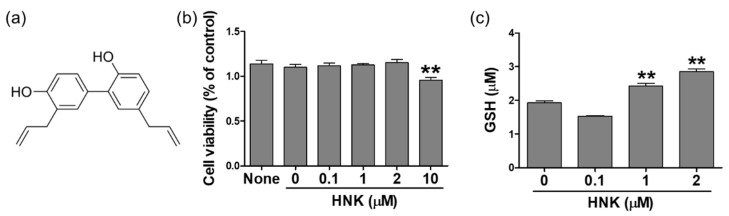
The structure of honokiol (HNK) and the cell viability and glutathione (GSH) content in human kidney-2 (HK-2) cells. (**a**) A chemical structure of honokiol. (**b**) Cells were treated with honokiol (0, 0.1, 1, 2, or 10 μM) for 24 h and MTT assays were performed. (**c**) Cells were treated with honokiol (0, 0.1, 1, or 2 μM) for 24 h and the GSH levels were determined. Values are expressed as the means ± S.E.M. of three independent experiments. ** *p* < 0.01 compared with the vehicle control.

First, we determined non-cytotoxic concentration of honokiol (the structure shown in Figure 1a) by performing MTT assay in HK-2 cells. The logarithmically growing cells were split and grown overnight, then treated with honokiol for 24 h at the indicated concentrations, and finally collected for following assays. Specifically, the cell viability was accessed in the cells treated with vehicle or honokiol at the concentration of 0.1, 1, 2, or 10 μM for 24 h. The results showed that cell viability was significantly reduced to 84% compared to control (100%) at 10 μM of honokiol in HK-2 cells (Figure 1b). To investigate whether honokiol treatment affects cellular redox homeostasis, we measured cellular levels of GSH, a major antioxidant that provides reducing equivalents for the glutathione peroxidase (GPx) reaction. The GSH levels were increased significantly in the cells treated with honokiol in a dose-dependent manner (Figure 1c). The GSH levels were increased by 1.26 and 1.48 at 1 and 2 μM of honokiol, respectively, compared to control. 

### 3.2. Honokiol Treatment Upregulated the Expression of GSH Biosynthetic Enzymes in HK-2 Cells

**Figure 2:**
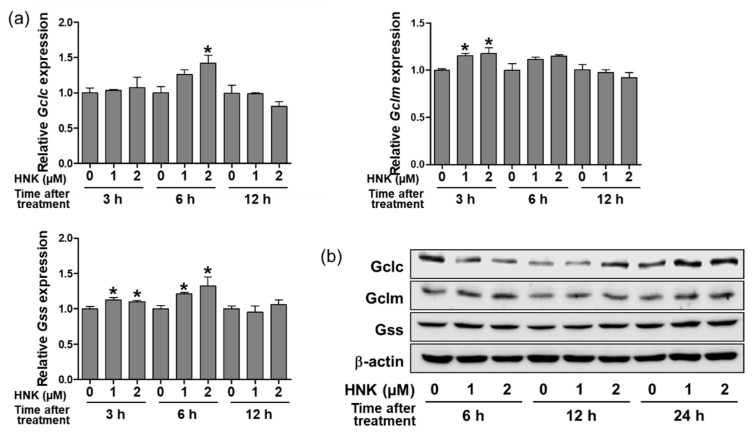
Honokiol increases the expression of GSH biosynthetic enzymes in HK-2 cells. (**a**) Cells were treated with honokiol (0, 1, or 2 μM) for 3, 6, or 12 h and the mRNA expression of Gclc, Gclm, and Gss was determined by real-time PCR analysis. Relative mRNA levels were normalized to those of GAPDH. (**b**) Cells were treated with honokiol (0, 1, or 2 μM) for 6, 12, or 24 h and the protein expression of Gclc, Gclm, and Gss was determined by Western blot analysis. Relative protein levels were normalized to those of β-actin. Values are expressed as the means ± S.E.M. of three independent experiments. * *p* < 0.05 significant compared with the vehicle control.

We investigated whether honokiol increases the biosynthesis of GSH through upregulating the expression of two key enzymes, glutamate cysteine ligase (Gclc and Gclm subunits) and glutathione synthetase (Gss). The relative mRNA levels of Gclc, Gclm and Gss were accessed in the cells treated with 1 or 2 μM of honokiol for 3, 6, or 12 h. The results showed that honokiol increased significantly the Gclc levels at 2 μM after 6 h, the Gclm levels at 1 and 2 μM after 3 h, the Gss levels at 1 and 2 μM after 3 and 6 h (Figure 2a). In addition, honokiol increased the protein levels of Gclc at 2 μM after 12 and 24 h, Gclm at 2 μM after 24 h; however, the level of Gss was not significantly changed after treatment (Figure 2b).

### 3.3. Honokiol-Induced Upregulation of Gclc Was Regulated by Nuclear Nrf2 Translocation in HK-2 Cells

**Figure 3:**
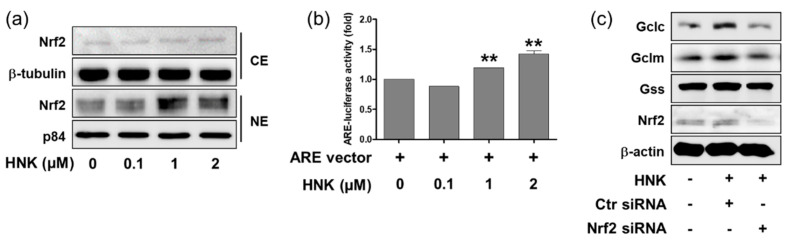
Honokiol increases nuclear translocation Nrf2 and ARE-luciferase activity in HK-2 cells. (**a**) Cells were treated with honokiol (0, 0.1, 1, or 2 μM) for 4 h and the cytosolic and nuclear fractionation were extracted and subjected to Western blot analysis. Nrf2 protein levels were normalized to those of β-tubulin and p84 in cytosolic and nuclear fractions, respectively. (**b**) Cells were transiently transfected with ARE-luciferase plasmid (1 μg) for 24 h, and treated with honokiol (0, 0.1, 1, or 2 μM) for 4 h. The ARE-luciferase activity was measured using a luciferase reporter assay system. (**c**) Cells were transiently transfected with control siRNA or Nrf2-specific siRNA (50 nM) for 24 h and treated with honokiol (2 μM) for 12 h. Then, cell lysates were subjected to Western blot analysis using anti-Gclc, anti-Gclm, anti-Gss or anti-β-actin antibodies. The + and − indicate the presence or absence of each treatment. Values are expressed as the means ± S.E.M. of three independent experiments. ** *p* < 0.01 significant compared with the vehicle control.

Many antioxidant genes including Nqo1, Gclc, Gclm, and HO-1 are activated transcriptionally by Nrf2. Thus, we investigated whether honokiol treatment stimulates the Nrf2-mediated transcriptional activity of Gclc, Gclm, and Gss in HK-2 cells. First, the cytoplasmic and nuclear Nrf2 expression was determined by Western blot analysis after subcellular fractionation of HK-2 cells; honokiol treatment increased significantly nuclear Nrf2 levels at the concentrations up to 1 μM (Figure 3a). Second, Nrf2-mediated ARE transcriptional activity was accessed by a luciferase reporter assay in HK-2 cells. Cells were transfected with the ARE-luciferase plasmid for 24 h and treated with 0.1, 1, or 2 μM of honokiol for 4 h. The results showed that honokiol increased significantly the ARE-luciferase activity at the concentrations up to 1 μM (Figure 3b). Third, we investigated whether honokiol-induced upregulation of Gclc and Gclm was blocked in Nrf2-silenced HK-2 cells. Cells were transfected with control or Nrf2 siRNA for 24 h and treated with 2 μM of honokiol for 12 h. The results showed that honokiol increased the Gclc and Gclm expression in the control siRNA-transfected cell; however, the expression was not changed in Nrf2 siRNA-transfected cells. In comparison, honokiol treatment affected the Gss expression in nether of control or Nrf2 siRNA-transfected cells (Figure 3c). Theses suggest that the honokiol-induced upregulation of Gclc and Gclm is through Nrf2-mediated transcriptional activation in HK-2 cells.

### 3.4. Honokiol Treatment Increased Nrf2 Transcriptional Activity through PI3K/Akt and PKC Signaling Pathway in HK-2 Cells

**Figure 4:**
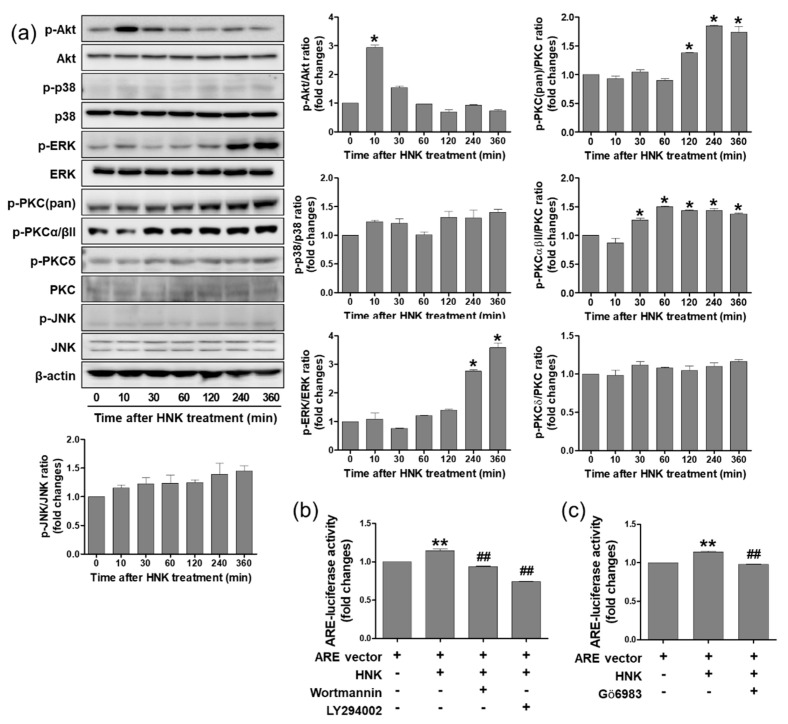
Honokiol regulates Nrf2 activation through phosphoinositide 3-kinase (PI3K)/Akt and protein kinase C (PKC) signaling pathway in HK-2 cells. (**a**) Cells were treated with honokiol (2 μM) for 0, 10, 30, 60, 120, 240, or 360 min. The cell lysates were subjected to western blot analysis using anti-p-Akt, anti-p-p38, anti-p-JNK, anti-p-ERK, anti-p-PKC (pan), anti-p-PKCα/βII, anti-p-PKCδ, and anti-β-actin antibodies. (**b**,**c**) Cells were transfected with ARE-luciferase plasmid (1 μg) and treated with inhibitors of Wortmannin (100 nM), LY294002 (10 μM), or Gö6983 (10 nM) for 30 min, prior to honokiol (2 μM). After 4 h incubation, the ARE-luciferase activity was measured by a luciferase reporter assay system. Values are expressed as the means ± S.E.M. of three independent experiments. * *p* < 0.05, ** *p* < 0.01 significant compared with the vehicle control. **^##^** *p* < 0.01 significant compared with honokiol alone.

The Nrf2 transcriptional activity has shown to be enhanced by signaling kinases, such as extracellular signal-regulated kinase (ERK), c-Jun N-terminal kinase (JNK), p38 mitogen-activated protein kinase (MAPK), phosphoinositide 3-kinase (PI3K)/Akt, and protein kinase C (PKC) in various cell types [18,19,20,21,22]. To determine whether honokiol activates Nrf2 through these kinases in HK-2 cells, honokiol (2 μM) was treated for varying times (0, 10, 30, 60, 120, 240, or 360 min) and the p-Akt, p-p38, p-JNK, p-ERK, and p-PKC levels were examined by Western blot analysis. While the p-p38 and p-JNK levels were not altered at honokiol treatment, the p-Akt and p-ERK levels were increased significantly at 10 min and 240–360 min after honokiol treatment, respectively. The p-PKC (pan) and p-PKC α/βII levels were increased gradually after honokiol treatment and maintained the enhanced levels until 360 min. The p-PKC δ levels were not altered at honokiol treatment (Figure 4a). To investigate whether PI3K/Akt and PKC signaling pathway is involved in Nrf2 transcriptional activity directly, the ARE-luciferase activity was measured after honokiol treatment in the presence of Wortmannin, LY294002 (specific inhibitors of PI3K/Akt), or Gö6983 (a pan-PKC inhibitor). The results showed that the honokiol-induced ARE-luciferase activities were significantly reduced by the inhibitors of PI3K/AKt and PKC (Figure 4b,c).

### 3.5. Honokiol Increases GSH Biosynthesis through PI3K/Akt and PKC Signaling in HK-2 Cells

**Figure 5:**
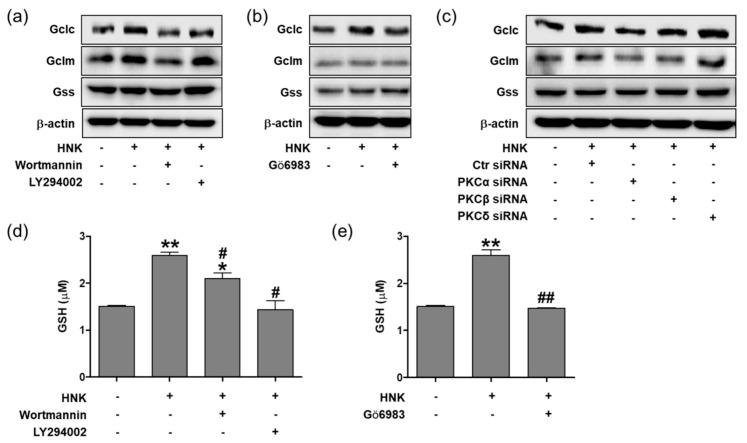
Honokiol regulates the biosynthesis of GSH through PI3K/Akt and PKC signaling pathway in HK-2 cells. (**a**,**b**) Cells were treated with inhibitors of Wortmannin (100 nM), LY294002 (10 μM), or Gö6983 (10 nM) for 30 min prior to honokiol (2 μM). After 12 h incubation, the cell lysates were subjected to Western blot analysis using anti-Gclc, anti-Gclm, anti-Gss, or anti-β-actin antibodies. (**c**) Cells were transiently transfected with specific RNAi for PKCα, PKCβ, or PKCδ, or control siRNA (100 nM) for 24 h, and then treated with honokiol (2 μM) for 12 h. The cell lysates were subjected to Western blot analysis using anti-Gclc, anti-Gclm, anti-Gss, or anti-β-actin antibodies. (**d**,**e**) Cells were treated with inhibitors of Wortmannin (100 nM), LY294002 (10 μM), or Gö6983 (10 nM) for 30 min prior to honokiol (2 μM). After 24 h incubation, the GSH levels were measured. Values are expressed as the means ± S.E.M. of three independent experiments. * *p* < 0.05, ** *p* < 0.01 significant compared with the vehicle control. **^#^** *p* < 0.05, **^##^** *p* < 0.01 significant compared with honokiol alone.

To investigate whether honokiol treatment increases the biosynthesis of GSH through PI3K/Akt and PKC signaling, the expression levels of biosynthetic enzymes, Gclc, Gclm, and Gss were analyzed after pretreatment of the specific inhibitors in HK-2 cells. After honokiol treatment, the Gclc and Gclm levels were not increased in the presence of Wortmannin, and the Gclc levels were not increased in the presence of LY294002 or Gö6983 (Figure 5a,b). In addition, the Gclc and Gclm levels were not increased after honokiol treatment in the cells transfected with siRNA specific to PKCα or PKCβ (Figure 5c), but were increased in the cells transfected with siRNA specific to PKCδ. Finally, cellular GSH content was determined in the presence of Wortmannin, LY294002, or Gö6983; the honokiol-induced GSH levels were decreased by these inhibitors (Figure 5d,e). These results suggest that honokiol increases the biosynthesis of GSH by activating Nrf2-mediated transcription of Gclc and Gclm through Akt/PI3K and PKC (particularly through PKCα and PKCβ, not through PKCδ) signaling in HK-2 cells. To investigate whether honokiol protects cells from the hypoxic condition, we measured the levels of GSH and malondialdehyde (MDA), an oxidative stress marker in H_2_O_2_ (600 μM)-treated cells after 2 μM of honokol treatment for 24 h. Honokol increased GSH levels and decreased MDA levels in H_2_O_2_-treated cells, compared to vehicle control (Supplementary Figure S1a,b). 

### 3.6. Honokiol Attenuates Renal Ischemia-Reperfusion Injury by Increasing GSH Biosynthesis

**Figure 6:**
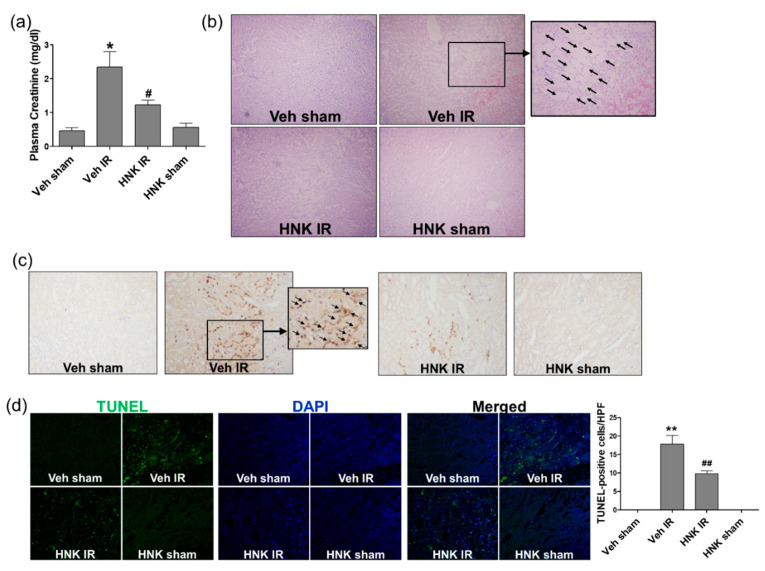
Honokiol attenuates the increase in plasma creatinine, tubular cell death and neutrophil infiltration induced by renal ischemia-reperfusion (IR) in mice. C57BL/6 mice were subjected to 25 min of bilateral renal ischemia. Honokiol (1 mg/kg) or vehicle was injected twice, 1 h prior to ischemia and 4 h after reperfusion. The blood and kidney tissues were collected at 24 h of reperfusion. (**a**) Plasma creatinine levels were measured to assess the degree of kidney injury. (**b**) Representative images of H&E staining of renal sections to evaluate tubular pathology. Arrows indicate necrotic area. (**c**) Representative images of polymorphonuclear leukocytes (PMN) staining of renal sections to evaluate neutrophil infiltration. (**d**) Representative images of terminal deoxynucleotidyl transferase dUTP nick end labeling (TUNEL) staining of renal sections to evaluate tubular apoptosis; TUNEL (green) merged with DAPI (blue). Values are expressed as the means ± S.E.M. of 4–8 mice from each group. * *p* < 0.05, ** *p* < 0.01 significant compared with Veh sham. **^#^** *p* < 0.05, **^##^** *p* < 0.01 significant compared with Veh IR.

To investigate whether honokiol protects the kidney from renal IR injury, mice were treated with honokiol and subjected to renal IR. Then, plasma creatinine was measured at 24 h of reperfusion (Figure 6a). Mice subjected to IR showed a significant increase in the plasma creatinine level, which was inhibited by honokiol treatment; Sham-operated mice treated with honokiol did not show any nephrotoxic feature. Histological analysis by H&E staining revealed that IR mice showed significant tubular necrosis and proteinaceous casts with increased congestion; however, honokiol treatment reduced renal necrosis and tubular injury. To determine whether honokiol treatment reduces renal inflammation and tubular apoptosis induced by renal IR, PMN and TUNEL staining was performed, respectively. Consistently, honokiol reduced the neurtrophil infiltration and tubular apoptosis induced by renal IR (Figure 6b,c).

**Figure 7:**
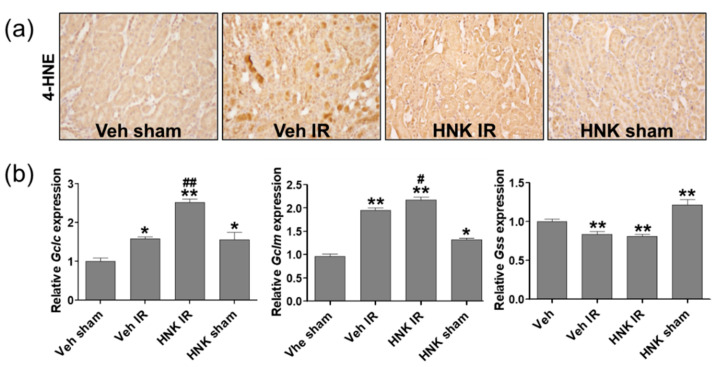

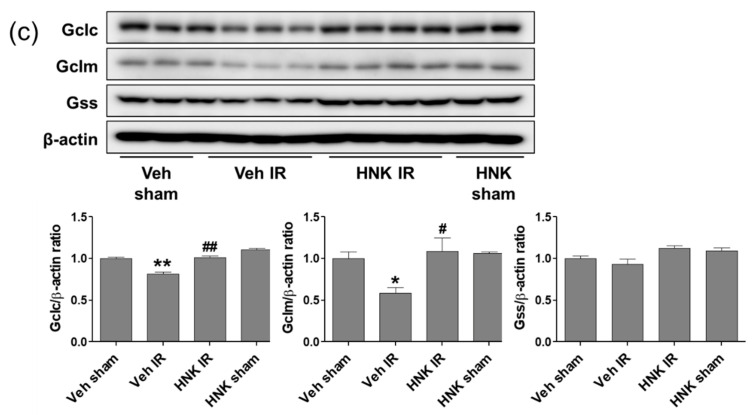
Honokiol attenuates the lipid peroxidation by increasing the expression of GSH biosynthetic enzymes in the kidney of IR-subjected mice. C57BL/6 mice were subjected to 25 min of bilateral renal ischemia. Honokiol (1 mg/kg) or vehicle was injected twice, 1 h prior to ischemia and 4 h after reperfusion. The kidney tissues were collected at 24 h of reperfusion. (**a**) Representative images of 4-HNE staining of renal sections to evaluate the lipid peroxidation. (**b**) The mRNA expression of Gclc, Gclm, and Gss was determined in kidney tissues by real-time PCR analysis. Relative mRNA levels were normalized to those of GAPDH. (**c**) The protein expression of Gclc, Gclm, and Gss was determined in the kidney tissue lysates using Western blot analysis. Relative protein levels were normalized to those of β-actin. Values are expressed as the means ± S.E.M. of 4–8 mice from each group. * *p* < 0.05, ** *p* < 0.01 significant compared with Veh sham. ***^#^*** *p* < 0.05, ***^##^*** *p* < 0.01 significant compared with Veh IR.

**Figure 8:**
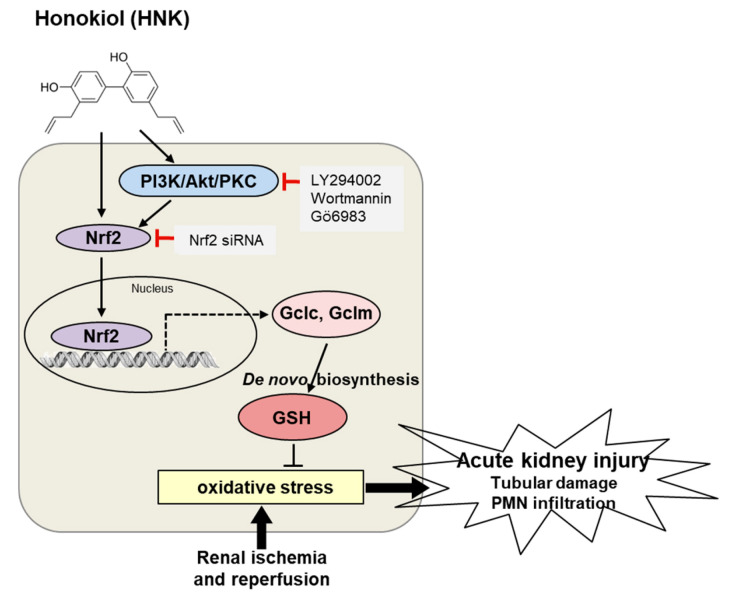
A proposed molecular mechanism of honokiol that protects the kidney against renal IR injury. Honokiol activates PI3K/Akt and PKC signaling, increasing nuclear translocation of Nrf2 for transcription of antioxidant genes including Gclc and Gclm. An increase of GSH exerts an antioxidant effect, attenuating IR injury. Solid, dashed, and bold arrows indicate signaling transduction pathways, gene transcription, and induced pathology, respectively. Different colors represent individual pathways or proteins.

To investigate whether honokiol treatment attenuates renal IR injury by increasing the biosynthesis of GSH, the mRNA expression of Gclc, Gclm, and Gss was examined in the kidney tissues of Veh sham, Veh IR, HNK IR and HNK sham mice (Figure 7a) by qPCR analysis. The protein expression was also examined by Western blot analysis (Figure 7b). Our results suggest that honokiol has a strong antioxidant effect by increasing the expression of GSH biosynthetic enzymes, Gclc and Gclm, attenuating the renal IR injury (Figure 8).

## 4. Discussion

The present study demonstrated the antioxidant effect of honokiol by upregulating the subunits of Gcl, a GSH biosynthetic enzyme in HK-2 cells as well as in the kidney exposed to IR injury. We suggest the underlying molecular mechanism that honokiol induces Nrf2 activation via PI3K/Akt and PKC signaling pathway, which was confirmed using pharmacological inhibitors and siRNA-mediated gene silencing. Thus, honokiol might provide an effective therapeutic strategy by reducing oxidative stress in patients with AKI as well as chronic kidney disease.

An imbalance of pro-oxidants and antioxidants occurs during the reperfusion phase of IR due to excessive ROS accumulation and impaired cellular redox systems [1]. A variety of endogenous antioxidants is present in the body to counteract external or internal sources of oxidants, and can be either enzymatic or non-enzymatic. The major enzymatic antioxidants are SODs, catalase and GSH peroxidase (GSH-Px); additional antioxidants are HO-1 and redox proteins (e.g., thioredoxins, peroxiredoxins, and glutaredoxins). Non-enzymatic antioxidants include GSH, vitamins C (ascorbate) and E (tocopherol), and carotenoids [32]. Particularly, GSH is the major soluble antioxidant and highly abundant in the cytoplasm. GSH has shown to recognize rapidly and have characteristic interactions with the hydroxyl radical (OH•) which is the most reactive species of ROS [22]. GSH also detoxifies hydrogen peroxide (H_2_O_2_) and lipid peroxides through the action of GSH-Px, protecting cell membrane from lipid oxidation; it also serves to convert vitamins C and E back to their active form [33,34].

Many natural compounds extracted from medicinal plants have shown antioxidant properties through scavenging ROS directly and activating endogenous antioxidant systems. GSH deficiency or metabolic dysfunction contributes to oxidative stress, playing a key role in aging and the pathogenesis of many diseases (e.g., neurodegenerative disease, cystic fibrosis, anemia, cancer, stroke, and diabetes) [15]. Several papers have reported the effects of polyphenols and flavonoids on enhancing activities of Gcl [35] and several antioxidant enzymes (e.g., GSH-Px, SOD, catalase, or GSH reductase) in cells stressed by UV irradiation or cytotoxin [36,37]. Interestingly, overexpression of Gclc subunits has shown to protect pancreatic islets from oxidative stress [38], or attenuate TNF-induced mitochondrial injury and apoptosis in mouse hepatoma cells [39]. Consistently, we demonstrated that honokiol upregulated the expression of Gcl subunits, alleviating IR-induced renal injury.

Recent review papers suggest that natural products activating Nrf2 have effectively attenuated oxidative stress, inflammation, and other distress in acute kidney injury caused by IR or nephrotoxin, and chronic renal diseases associated with diabetes, hypertension, and insulin resistance [40,41]. The transcription of many antioxidant enzymes is activated by Nrf2 binding on the ARE sequence, and the process of Nrf2 degradation or nuclear translocation is regulated by pharmacological active phytochemicals [42]. Quercetin or onion extract has shown to increase GSH levels in COS-1 and HepG2 cells by activating Gcl transcriptionally via the ARE [43,44]. In fact, Nrf2 plays as a master redox switch by the induction of many stress-responsive and cytoprotective enzymes. Certain phytochemicals have reported to target Keap1 thereby stabilizing Nrf2, or activate signaling kinases in the upstream of Nrf2 to facilitate nuclear localization [45]; activation of several upstream kinases, such as MAP kinases, PI3K/Akt, PKC, and casein kinase-2 have been demonstrated [46,47,48,49,50]. We also demonstrated that honokiol activated Nrf2 activation through activation of PI3K/Akt and PKC signaling.

Honokiol has traditionally been used to treat various diseases in China and East Asia countries and many research papers reported its beneficial effects and underlying mechanisms. Honokiol has shown anti-inflammatory, anti-cancer, anti-bacterial, neuroprotective properties under oxidative stressed conditions, such as LPS-induced hepatotoxicity, collagen (CII)-induced arthritis, bacterial infection, or hydroxydopamine-induced neurotoxicity [51,52,53,54,55]. Particularly, in our interest, honokiol has reported its renoprotective effect. Honokiol treatment improves animal survival of septic rats and reduces oxidative stress, inflammatory cytokines, and nitric oxide (NO) in sepsis-associated acute kidney injury (AKI) [29]. Honokiol also protects against renal IR injury by increasing SOD and catalase and suppressing oxidative stress, inducible NO synthase and inflammation in rats [30]. Honokiol protects H_2_O_2_-induced apoptosis of mouse podocytes by reducing the expression of caspase-3 and caspase-9 via Akt and Erk1/2 signaling [56]. Here, we found that honokiol upregulated cellular GSH by upregulating its biosynthesis through Nrf2 activation via PI3/Akt and PKC signaling. The protective role of honokiol in IR has been reported in our and previous studies, but further studies on the precise molecular mechanisms are needed.

In conclusion, the present study demonstrated that honokiol increased GSH, a major cellular antioxidant, by upregulating the expression of biosynthetic enzyme subunits, Gclc and Gclm, through Nrf2 activation, where PI3K/Akt and PKC signaling pathways were involved. Thus, honokiol has a therapeutic potential that the activation of Nrf2 results in induction of multiple antioxidant and cytoprotective genes, which alleviates oxidative stress, inflammation, and other distress in acute IR and chronic renal injury, as well as associated diseases.

## Figures and Tables

**Table 1:** Primers used in this study.

Genes	Primers (5′-3′)
Human Gclc	Forward: CCCAAACCATCCTACCCTTT
	Reverse: CATGTTGGCCTCAACTGTATTG
Human Gclm	Forward: CCTGTTCAGTCCTTGGAGTTG
	Reverse: CCTCCCAGTAAGGCTGTAAATG
Human Gss	Forward: CCTGTTCAGTCCTTGGAGTTG
	Reverse: CCTCCCAGTAAGGCTGTAAATG
Human GAPDH	Forward: GGTGTGAACCATGAGAAGTATGA
	Reverse: GAGTCCTTCCACGATACCAAAG
Mouse Gclc	Forward: CATCGACCTGACCATCGATAAG
	Reverse: AGGGTGAGTGGGTCTCTAATAA
Mouse Gclm	Forward: GTATCAGTGGGCACAGGTAAA
	Reverse: CGGGTCATTGTGAGTCAGTAG
Mouse Gss	Forward: ACCTTTGCTGGCCTCTATTC
	Reverse: TTGTTACCTCCACCCTCTCT
Mouse GAPDH	Forward: GTGGCAAAGTGGAGATTGTTG
	Reverse: TTGACTGTGCCGTTGAATTTG

## Supplementary Materials

The following are available online at https://www.mdpi.com/2227-9059/8/9/352/s1, Figure S1: Honokiol attenuates the lipid peroxidation by increasing the expression of GSH biosynthetic enzymes in H_2_O_2_-treated HK-2 cells.
