# Supplementary material for: Honokiol Protects the Kidney from Renal Ischemia and Reperfusion Injury by Upregulating the Glutathione Biosynthetic Enzymes
Source: Biomedicines. 2020 Sep 15;8(9):352. doi: 10.3390/biomedicines8090352 (PMC7555803; doi:10.3390/biomedicines8090352)
Supplement: Supplementary file 1 [file biomedicines-08-00352-s001.pdf]

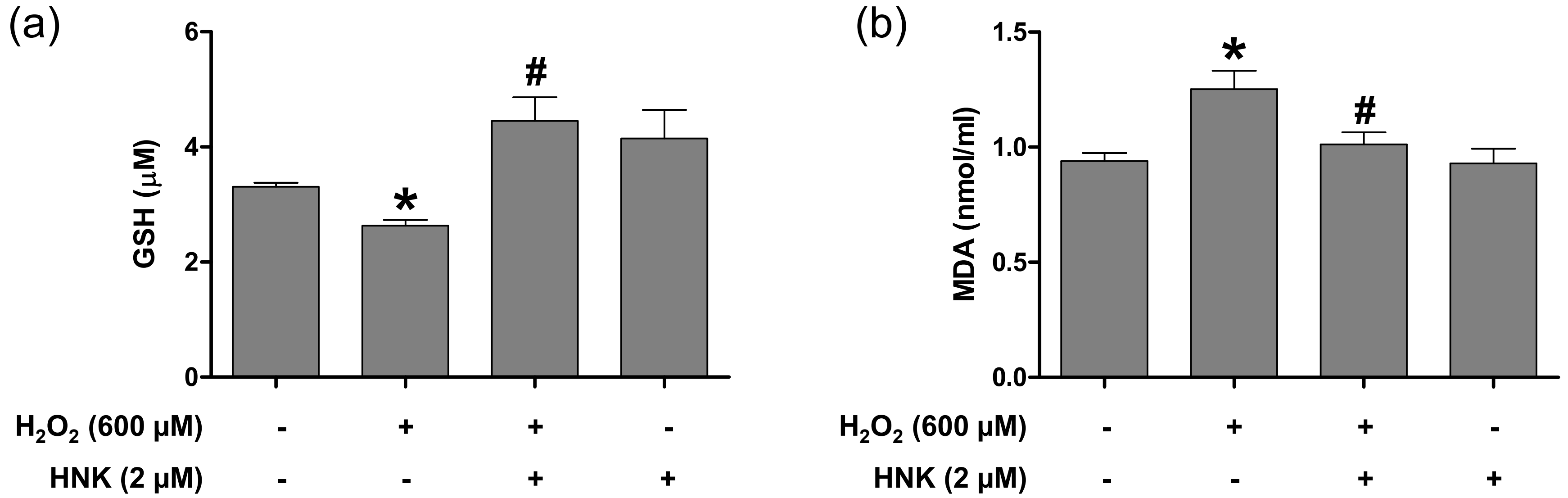

Supplementary figure S1. Honokiol attenuates the lipid peroxidation by increasing the expression of GSH biosynthetic enzymes in H_2O_2-treated HK-2 cells. Cells were treated with H_2O_2 for 24 h to induce oxidative damage, and then glutathione (GSH) (a) and malondialdehyde (MDA) (b) levels were determined. Honokiol (2 μM) was treated 1 h prior to H_2O_2 treatment. Values are expressed as the means ± S.E.M. *$P<0.05$ significant compared with the Vehicle control. #P<0.05 significant compared with the Vehicle+H_2O_2.
